# *Announcement*: U.S. Antibiotic Awareness Week — November 13–19, 2017

**DOI:** 10.15585/mmwr.mm6644a9

**Published:** 2017-11-10

U.S. Antibiotic Awareness Week is an annual observance to raise awareness about antibiotic resistance and the importance of appropriate antibiotic prescribing and use. This year’s observance coincides with the release of CDC’s updated educational initiative “Be Antibiotics Aware: Smart Use, Best Care,” and aims to engage health care professionals, advocacy groups, for-profit companies, state and local health departments, professional societies, the general public, the media, and others in efforts to improve antibiotic prescribing and use across all health care settings. This observance coincides with the World Health Organization’s World Antibiotic Awareness Week and European Antibiotic Awareness Day on November 18.

Antibiotics save lives. When a patient needs antibiotics, the benefits outweigh the risks for side effects or antibiotic resistance. However, antibiotic resistance is one of the most urgent threats to the public’s health. Each year in the United States, approximately 2 million persons are infected with antibiotic-resistant bacteria, and approximately 23,000 die as a result.* Helping health care professionals improve the way they prescribe antibiotics and improving the way patients take antibiotics helps keep everyone healthy now, helps fight antibiotic resistance, and ensures that lifesaving antibiotics will be available for future generations.

Preventing antibiotic-resistant infections and protecting the nation’s health by improving antibiotic prescribing and use is a CDC priority. Additional information about “Be Antibiotics Aware” during U.S. Antibiotic Awareness Week is available at https://www.cdc.gov/antibiotic-use.

## Footnotes

* https://www.cdc.gov/drugresistance/index.html.

